# The hidden identity of faces: a case of lifelong prosopagnosia

**DOI:** 10.1186/s40359-019-0278-z

**Published:** 2019-01-22

**Authors:** Martin Wegrzyn, Annika Garlichs, Richard W. K. Heß, Friedrich G. Woermann, Kirsten Labudda

**Affiliations:** 1 Department of Psychology, Bielefeld University, Bielefeld, Germany; 2 Bethel Epilepsy Center, Mara Hospital, Bielefeld, Germany

**Keywords:** Developmental prosopagnosia, Object recognition, Face perception, Configural processing, fMRI

## Abstract

**Background:**

Not being able to recognize a person’s face is a highly debilitating condition from which people with developmental prosopagnosia (DP) suffer their entire life. Here we describe the case of J, a 30 year old woman who reports being unable to recognize her parents, her husband, or herself in the mirror.

**Case presentation:**

We set out to assess the severity of J’s prosopagnosia using tests with unfamiliar as well as familiar faces and investigated whether impaired configural processing explains her deficit. To assess the specificity of the impairment, we tested J’s performance when evaluating emotions, intentions, and the attractiveness and likability of faces. Detailed testing revealed typical brain activity patterns for faces and normal object recognition skills, and no evidence of any brain injury. However, compared to a group of matched controls, J showed severe deficits in learning new faces, and in recognizing familiar faces when only inner features were available. Her recognition of uncropped faces with blurred features was within the normal range, indicating preserved configural processing when peripheral features are available. J was also unimpaired when evaluating intentions and emotions in faces. In line with healthy controls, J rated more average faces as more attractive. However, she was the only one to rate them as less likable, indicating a preference for more distinctive and easier to recognize faces.

**Conclusions:**

Taken together, the results illustrate both the severity and the specificity of DP in a single case. While DP is a heterogeneous disorder, an inability to integrate the inner features of the face into a whole might be the best explanation for the difficulties many individuals with prosopagnosia experience.

## Background

J is a 30 year old woman who approached our research department and asked to be examined for possible prosopagnosia. According to her self-report, J has a lifelong inability to recognize the identity of others from their faces. This includes an inability to recognize her parents, her husband, and (under certain conditions) herself in the mirror.

The first case of prosopagnosia present from early childhood, now called developmental prosopagnosia (DP), was described by McConachie in 1976 [1]. Unlike the prosopagnosia cases reported before [2, 3], McConachie’s patient showed no signs of brain damage that could explain the condition. It took 20 years until a second case of DP was reported [4], and hence the condition was considered to be very rare. However, with increased awareness of prosopagnosia in public, more people reporting symptoms of “face blindness” have come forward [5]. Today it is estimated that around 2% of the general population presents with DP [6, 7]. Similar to other people with lifelong prosopagnosia [8, 9], J reports that she was oblivious to the nature of her condition for the vast majority of her life. *“It simply never occurred to me that one could recognize people only by their face”,* J told us. A few years ago she was watching a TV talk show, in which one of the guests was interviewed about his prosopagnosia. J describes this moment as the one in which she immediately knew that she must have the same condition.

Because a researcher on the show was looking for participants to enroll in a study concerned with hereditary forms of prosopagnosia (cf. [10, 11]), J asked her family members whether they had the same problem as her. No one in J’s family identified with the symptoms she described, which makes it likely that J has a non-hereditary form of the condition [12, 13]. Therefore, J could not enroll in the study advertised on television and instead visited neurologists and neuropsychologists, who in turn were unable to diagnose her with prosopagnosia, as neither diagnostic criteria [14, 15] nor test instruments [7] are so far clinically established.

However, some hallmarks of DP have been agreed upon in the research literature: The main deficit is an inability to recognize individuals by their faces, but preserved ability to recognize them by non-face information such as voice, gait or clothes [16]. The onset of the condition is at least in early childhood, and the deficits are sustained throughout life [17]. Furthermore, there is no evidence for acquired brain damage that could explain the symptoms [18].

Regarding the age of onset, J reports that she cannot remember a time when she was able to recognize people by their faces and recalls conspicuous behavior she showed as far back as grade school. Back then, she would spend most of her time in the schoolyard with her best friend, who would constantly have to tell her the names of classmates they passed on the schoolyard, and point out people they knew. J told us that back then she did not suspect she was somehow impaired, but rather thought that her friend was simply exceptionally good at recognizing people. Also, it was not obvious to J what information her friend was using to recognize people. It did not occur to her that it was their faces which gave away other people’s identities.

**Fig. 1:**
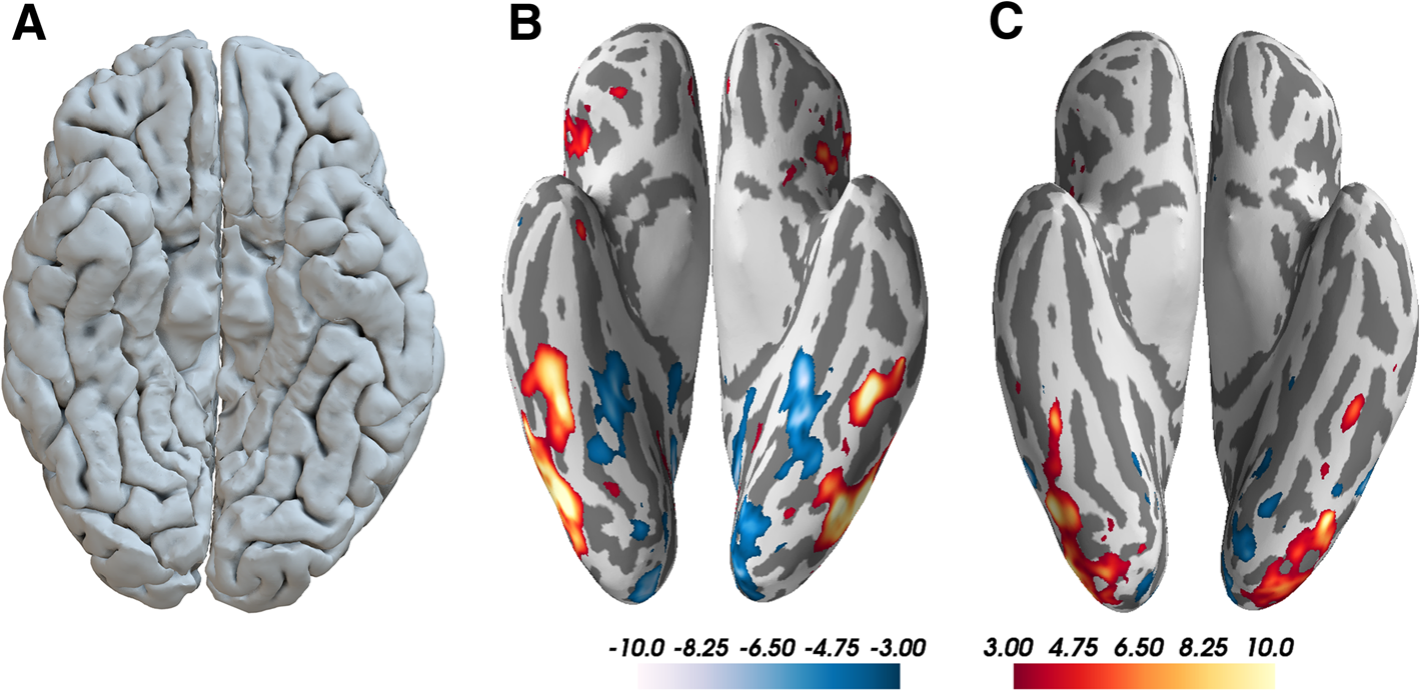
Results of structural and functional brain imaging. **a** inferior view of the cortical surface reconstructed from high-resolution structural MRI, depicting that the fusiform gyri show no sign of being abnormal; **b** results for the “faces vs. landscapes” fMRI localizer task; **c** results from the “faces vs. hands” fMRI localizer task. fMRI results are shown on the inflated cortical surface, with face activity shown in warm colors and activity for the respective control condition shown in cool colors. Results are thresholded at t = 3. For both paradigms, strong bilateral activity can be seen for the face condition, in the lateral parts of the fusiform gyri. The anterior clusters in the fusiform gyri most likely correspond to the “fusiform face area” and the posterior clusters in the lateral occipital cortex most likely correspond to the “occipital face area”. Unthresholded normalized surface maps are available online: https://neurovault.org/collections/4017/

Previous to visiting our lab, J had two clinical MRIs which were diagnosed as asymptomatic. This diagnosis was confirmed by our own MRIs, which do not show any abnormalities on a macroanatomical scale, including the fusiform gyri (Fig. 1a). Areas in the lateral part of the fusiform gyrus are important for face processing [19] and these are the areas damaged in the acquired forms of prosopagnosia [20] However, they have been shown to be intact even in severe cases of DP [21–24].

To characterize both the specificity and the severity of J’s self-reported difficulties with faces, we asked her to perform a number of different tests. These were designed to delineate deficits restricted to recognizing the identity of faces from more global deficits in processing non-identity information in faces and other objects. Furthermore, tests with different degrees of difficulty were used and J’s performance was compared relative to matched healthy controls or to normative data, whenever feasible. Finally, to better understand the possible cognitive mechanisms which underlie DP, we designed experiments which allowed us to evaluate how successfully J uses different face processing strategies, such as configural processing [25], where information in the face needs to be integrated into a whole.

### Case presentation

#### Object recognition and ventral stream functions

DP is a condition of varying specificity and particularly the early cases were impaired in more general object recognition as well [1, 4]. Later, very “pure” cases, restricted to deficits in recognizing only the identity of faces were reported [23, 26, 27]. To exclude a more general form of agnosia, we performed a screening with living as well as non-living objects (cf. methods section), on which J made no mistakes. In two fMRI tasks with faces, hands and landscapes as stimuli, J showed prototypical activity in the lateral fusiform gyrus for faces (Fig. 1 b, c) and activity in medial parts of the ventral stream for landscapes (Fig. 1b). These are the patterns which are predicted by the standard models of object processing [19] and indicate that J’s abilities to differentiate between faces and non-face objects are unimpaired. Such normal activation patterns in the fusiform gyrus for faces, lateral to the mid-fusiform sulcus [28], have been reported in previous cases with DP [29–31]. However, from discussions with J we learned that faces as a superordinate category might pose a challenge for her when forming or accessing internal representations. For example, she often retains vivid recollections of her dreams, but they do not feature faces: “*Whenever there is a person, instead of the face there is just a placeholder of some kind”.* When reading fiction, J claims that she always imagines the actions of the people described from a first-person perspective, which obviates imagining what the person performing that action might look like. Deficits in imagery for faces have also been found in some previous studies on DP [32, 33].

Furthermore, J complained to us about problems with body perception, specifically correctly identifying her left and right hand. While J is unequivocally right handed according to the Edinburgh handedness questionnaire [34], she claims that she often uses the *“wrong”* (i.e. left) hand when initiating a movement and then has to correct herself and switch hands. In patients with damage to the fusiform gyrus, similar problems with body perception have been reported [35]. Accordingly, J’s performance on the Bergen Right-Left Discrimination Test [36] was two standard deviations below average (cf. Table 1), while her performance on a more general visuo-spatial task (subtests six to eight of the LPS-2; [37]) was poor but still within the normal range (percentile 16). This might indicate that some body-specific visuo-spatial functions, which are neuroanatomically located close to the primary face processing areas, can also be impaired in certain cases of DP.

**Table 1:** Overview of J’s performance for all major tasks

Domain	Test	% correct	percentile	z	t	df	p
Face recognition	CFMT 1	100	73	0.61	0.59	26	.561
	CFMT 2	37	< 1	−3.53	−3.40	26	.002
	CFMT 3	33	1	−2.33	−2.25	26	.033
	Famous familiarity	31	< 1	−3.60	−3.47	26	.002
	Famous context	58	1	−2.40	−2.32	26	.029
	Famous naming	14	1	−2.39	−2.31	26	.029
Face evaluation	Emotion recognition	88	87	1.15	1.08	16	.294
	Eyes test	72	21	−0.81	−0.80	49	.425
	Attractiveness	77	64	0.37	0.36	45	.720
	Likability	44	< 1	−3.36	−3.29	45	.002
Visuo-spatial	BRLD-A	33	1	−2.52	−2.52	173	.013
	BRLD-B	38	2	−2.02	− 2.01	173	.046
	LPS2-Visual	48	16	−1.00	−1.00	128	.321

CFMT: Cambridge Face Memory Test [38]; Eyes test: Reading the mind in the eyes; BRLT: Bergen Right-Left Discrimination Test [36]; LPS2-Visual: Leistungsprüfsystem 2 (“performance test system”), test of visuo-spatial skills [37]

#### Recognition of unfamiliar faces

J reports that her condition is especially impairing when she has to learn new faces, as was the case when she held a job in sales and distribution: “*If you have a job where you sit in your office and people come to you at previously appointed times, it is easy. But if you have to actively approach people, go to their offices, or make small talk in the hallway, you can’t do it if you don’t know who is who”.* Her inability to recognize customers and colleagues therefore had many repercussions, even leading to the loss of some jobs.

**Fig. 2:**
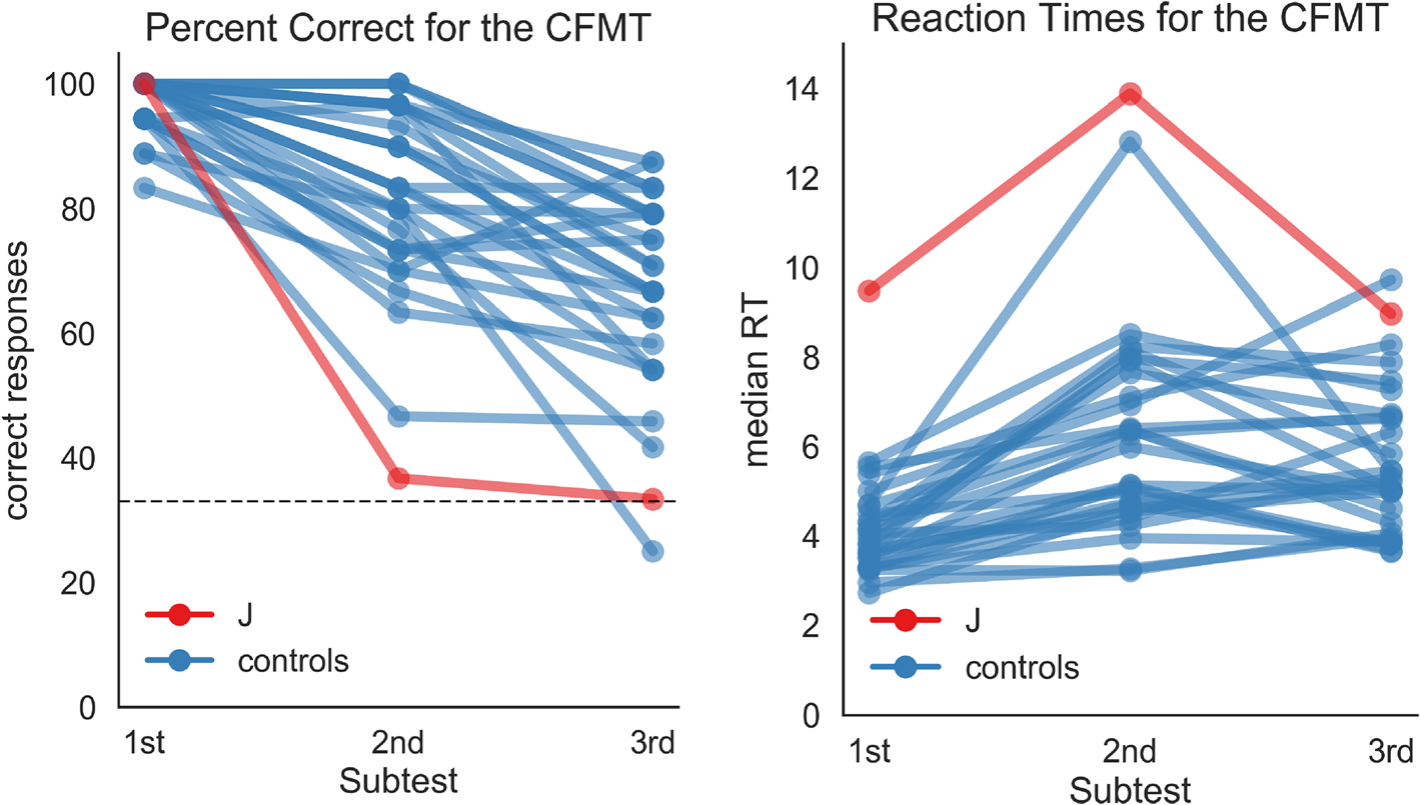
Results of the Cambridge Face Memory Test (CFMT). J shows perfect performance when one face needed to be remembered and recognized (1st part), but is among the slowest and most inaccurate participants when six faces had to be remembered and recognized (parts 2 and 3). Averaged over all three parts of the CFMT, J answered 37 out of 72 items correctly (51%). On average, the control participants answered 59 items correctly, (SD = 7), so to be within 2 SDs at least 45 hits are needed

To test J’s ability to learn and recognize new faces, we used the Cambridge Face Memory Test (CFMT; [38]), which is the most established test in DP research, and is considered most promising as a clinical diagnostic instrument [18, 39]. It hides hair and other external features in the pictures and does not show faces simultaneously but consecutively, so feature matching is not as easily possible [38]. In the first part of the test, one face is shown for 2 s and after a brief pause 3 faces appear, the learned face being one of them. In this part, J scored a perfect 100% correct when trying to recognize the learned face (Fig. 2). Afterwards, she explained to us that for each face she tried to find one or two characteristic features, verbalize them for herself and then search for them in subsequent pictures (e.g. “*elongated chin”,* “*fair eyes”, “chubby cheeks”*).

In the next two parts of the CFMT, six faces are presented simultaneously for 20 s to study and then have to be recognized in subsequent arrangements of 3 faces, of which one is from the learned set. This task is either performed under normal viewing conditions (part 2) or under conditions where faces are strongly degraded by noise (part 3). In both of these tasks, J performed at chance level (Fig. 2). When asked afterwards what she found difficult, J reported that her strategy of finding one characteristic feature for each face breaks down once many faces need to be learned at the same time, as no single feature is unique to each face any more.

Given this pattern of results (perfect performance on the first part of the CFMT), we decided against adding a dedicated face matching task like the Cambridge Face Perception Test [7]. Instead, we tried to further narrow down J’s problems with face memory.

#### Recognition of familiar faces

While remembering faces seen only on one occasion is a difficult task per se, people with DP are also unable to recognize individuals they have known for years [4, 23, 24, 40]. Similarly, J reported several occasions on which she passed her husband on the street without recognizing him. On another occasion, J passed a large wall of mirrors in a department store and, for a brief moment, mistook her own reflection for somebody mimicking her movements. Also, J noted that when she looks at pictures of herself, e.g. taken on a vacation, she is surprised every time she sees a picture of herself. “*I cannot remember what I look like”,* she told us.

Despite being unable to recognize her husband or herself, J claims that there are a number of famous people she can recognize by their faces with high certainty. Studies in which participants with DP were asked to recognize famous faces have shown that they are significantly worse than controls in these tasks. However, their absolute scores are surprisingly high, as they usually can still identify around 30–40% of the faces correctly [16, 41]. One explanation is that those famous faces for which there has been more exposure might be easier to recognize [16]. However, this contradicts the observation that most cases of DP have difficulties in recognizing family members and themselves, despite lifelong exposure [8, 23, 40, 42].

To test J’s ability to recognize famous faces and directly address the role of exposure, we asked her to provide us with a list of people she thinks she can recognize from looking at their face alone. The list J provided consisted of 14 celebrities, including Angela Merkel, Barack Obama, some (but notably not all) actors from the shows “How I Met Your Mother”, “House” and “Law and Order”, as well as some other politicians and actors. In our final experiment, there were 42 famous people (5 images per person), including the ones from J’s list, actors on the same shows who were not on the list, and other famous people (cf. Table 2). All pictures were gray-scaled and prepared so that only inner features were visible. The task for J was to first decide if she knew the person (familiarity) then if she could pick the context from which the person might be familiar and finally to write down the person’s name (either the actual name or, in the case of actors, the name of the character they play).

**Table 2:** Detailed results of the famous face experiment for J

Known For/As	Name	On List	Known	Number Familiar	Number Names	Mistakes
HIMYM	Alyson Hannigan (Lily)	yes	yes	5	5	
	Jason Segel (Marshall)	yes	yes	4	4	
	Neil Patrick Harris (Barney)	yes	yes	4	3	
	Josh Radnor (Ted)	no	yes	0	0	
	Cobie Smulders (Robin)	no	yes	2	0	Mariska Hargitay
	Cristin Milioti (Tracy)	no	no	1	0	Audrey Hepburn
House	Robert Sean Leonard (Wilson)	yes	yes	5	5	
	Hugh Laurie (House)	yes	yes	4	4	
	Omar Epps (Eric Foreman)	no	yes	3	3	
	Lisa Edelstein (Lisa Cuddy)	no	yes	0	0	
Law & Order	Ice-T (Odafin “Fin” Tutuola)	no	yes	5	5	
	Mariska Hargitay (Liv)	yes	yes	3	3	
	Dann Florek (Donald Cragen)	no	yes	1	1	
Big Bang Theory	Jim Parsons (Sheldon Cooper)	no	yes	1	0	Wilson
	Kaley Cuoco (Penny)	no	yes	0	0	
Bones	Emily Deschanel (“Bones”)	yes	yes	4	4	a Spice Girl
Musician	Britney Spears	no	yes	3	3	
	Elvis Presley	no	yes	4	0	
	Beyonce	no	yes	0	0	
	Shakira	no	yes	0	0	
	Taylor Swift	no	no	0	0	
	Usher	no	no	0	0	
Movie Actor	Will Smith	yes	yes	5	5	
	Leonardo DiCaprio	no	yes	5	4	Brad Pitt
	Tom Cruise	no	yes	4	4	
	Nicole Kidman	yes	yes	3	3	
	George Clooney	no	yes	1	0	Charlie Sheen
	Mila Kunis	no	yes	0	0	
	Benedict Cumberbatch	no	no	1	0	
	Emma Watson	no	no	0	0	
Politician	Barack Obama	yes	yes	5	4	
	Angela Merkel	yes	yes	4	4	
	Sigmar Gabriel	no	yes	2	1	
	Sahra Wagenknecht	no	yes	0	0	
	Ursula von der Leyen	no	yes	0	0	
Sports-person	Joachim Loew	no	yes	5	4	Wilson
	Bastian Schweinsteiger	no	yes	0	0	
TV Host	Guenther Jauch	yes	yes	4	4	
News-anchor	Katja Burkard	yes	yes	0	0	
	Caren Miosga	yes	yes	2	0	Kate Winslet
	Jan Hofer	no	yes	0	0	
	Judith Rakers	no	yes	0	0	

J’s responses for the part of the famous face test where only inner features are shown. “Recognizable” refers to whether J thinks that she can usually recognize that person based on the face. “Known” refers to whether J said she knew who the person is. “Familiarity” refers to how often J reported a feeling of familiarity with the face, out of 5 trials. “Naming” refers to the number of times J could correctly name the person (out of 5). “Mistakes” lists any incorrect naming responses given by J. HIMYM: “How I Met Your Mother”

**Fig. 3:**
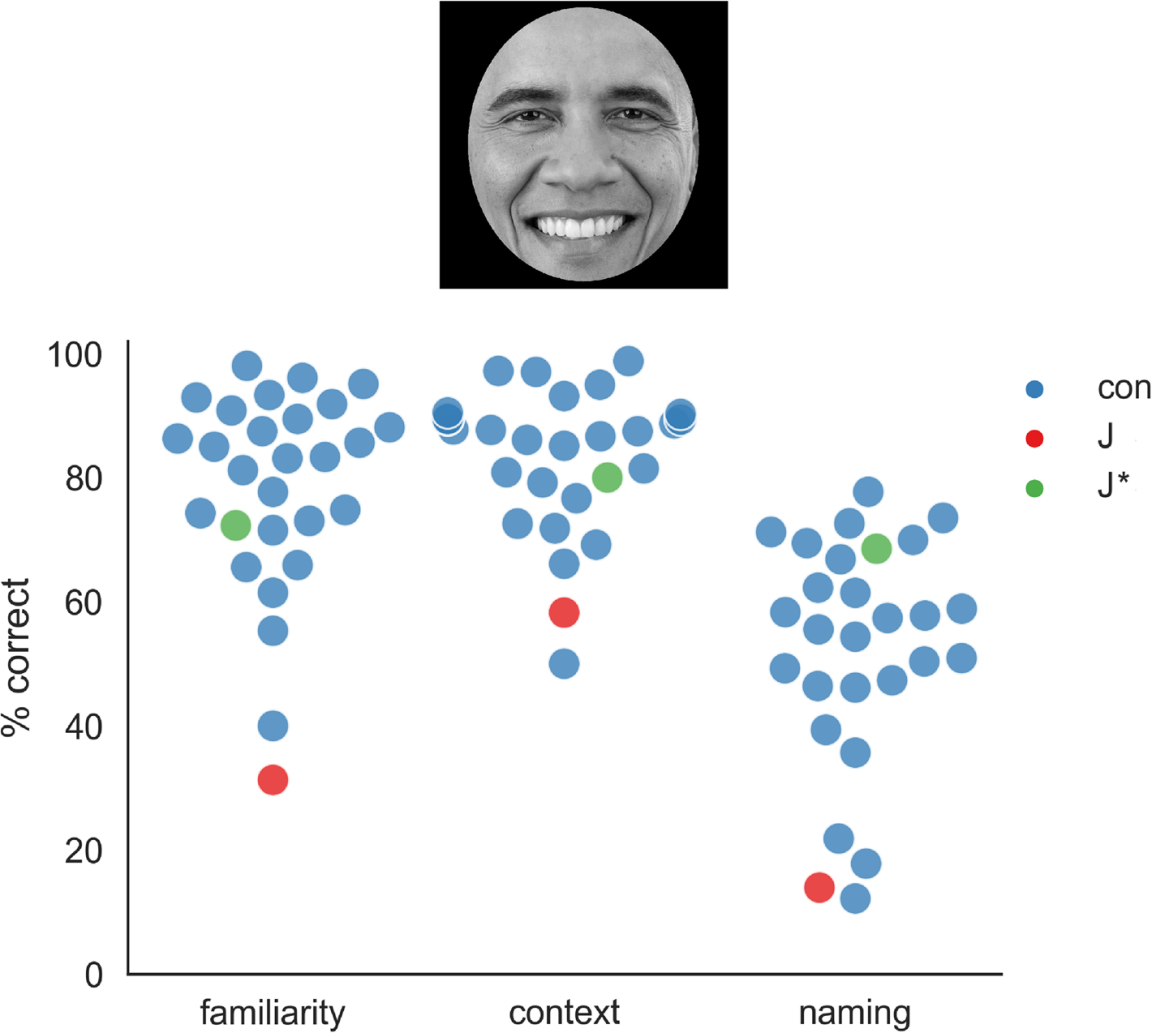
Results for the famous faces task. Face stimuli were grey-scaled and cut out with an ellipse so that only inner features were visible. For each face, three questions had to be answered: is the face familiar? (yes/no); from what context might that person be known? (politician, actor, musician, athlete, TV host); what is the name of the person? Results of the control participants (con) are shown in blue. J’s results for famous faces she did not preselect are shown in red and J’s results for faces which she previously picked as ones she thinks she can reliably recognize are shown in green (J*). Picture of Barack Obama is in the public domain (https://commons.wikimedia.org/wiki/File:President_Barack_Obama.jpg)

J’s performance was below the normal range for familiarity, context and for naming (Fig. 3, Table 1), indicating a clear deficit in recognizing familiar faces. However, her performance was average or above-average when only considering the faces on the list she prepared. Regarding free naming, J reached only 14% correct for famous people not on her list, but performed 69% correct for the famous faces who were on her list. This illustrates that J has good introspection into her abilities: there are faces that she can recognize by their inner features alone with high reliability. For example, J successfully recognized the characters Lily (5 out of 5), Marshall (4/5) and Barney (3/5) from the sitcom “How I Met Your Mother”, who were all previously on her list. The other two main protagonists Ted and Robin from the same show were not on her list and both were not recognized even once (both 0/5), although J should have had roughly the same amount of exposure to them.

To get a better understanding of how J performed that task, we later asked her to try and verbalize what makes recognizing certain faces easy. Regarding Ms. Merkel (4/5 correct namings), J pointed out that Ms. Merkel seems to have a “*characteristic facial expression that is kind of stiff”.* Regarding Mr. Obama (4/5 correct namings), J said that he is easy to recognize due to his “*freckles”,* a subtle feature which most observers (including the authors) probably do not notice but which prove useful for observers who heavily rely on recognizing individuals by single features.

#### Featural vs. configural processing

**Fig. 4:**
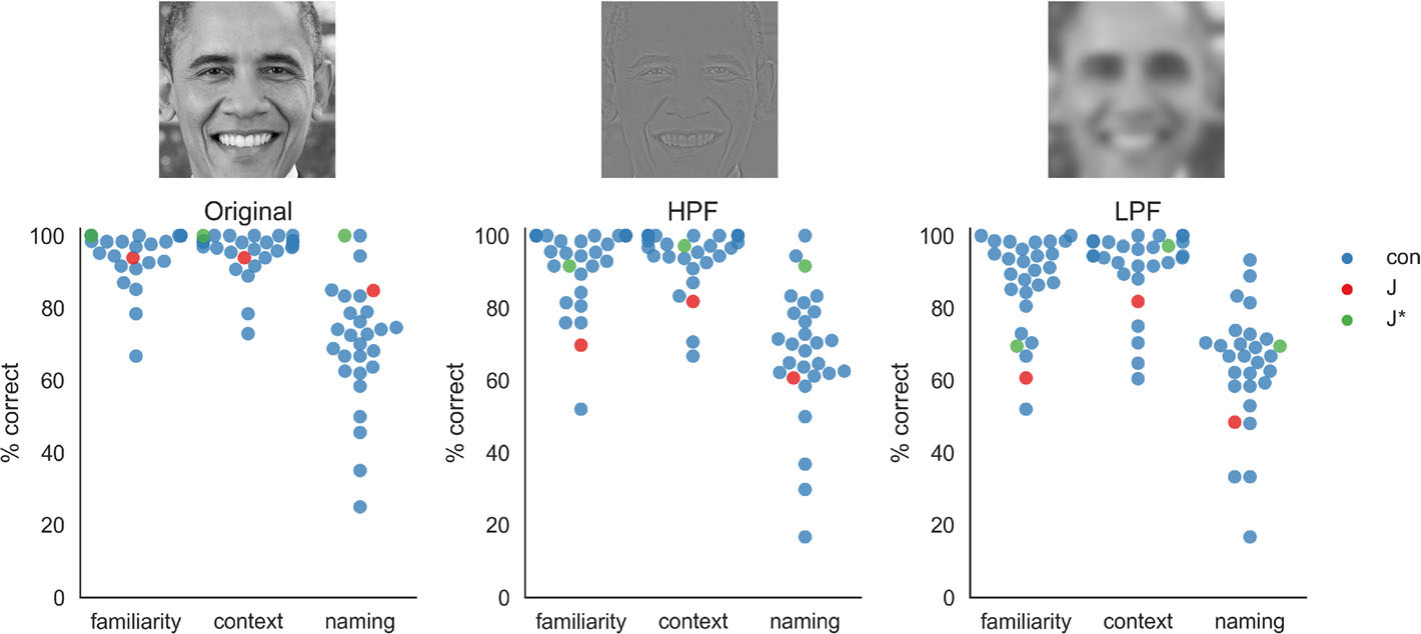
Results for the filtered famous faces. Face stimuli were all shown in grey-scale and with peripheral features visible. The “original” condition consists of stimuli with no further manipulation; the high-pass filtered (HPF) version consists of faces which were filtered so that the edges of features are emphasized; the low-pass filtered (LPF) version consists of faces which were smoothed with a Gaussian kernel so that features are blurred and featural processing of faces is not possible. Each face has to be rated regarding familiarity, context and the person’s name has to be given. Results of the control participants are shown in blue (con), J’s results for famous faces she did not preselect are shown in red and J’s results for faces which she previously listed as being recognizable are shown in green (J*). Except for the familiarity ratings on HPF and LPF faces, where J’s performance is 2 SD below the controls, her performance on all tasks and for both preselected and not preselected faces was within the normal range. Picture of Barack Obama is in the public domain

To better understand how J uses the features of the face compared to their configuration (so-called holistic or configural processing; [25]), we modified the famous faces task to show low-pass and high-pass filtered images. When applying a low-pass filter (Fig. 4), a single blurred feature, for example an eye, cannot be recognized as such in isolation, but only in the context of the whole face. Hence, using low-pass filtered images in recognition tasks is useful to test the integrity of configural face processing by preventing single-feature analyses. As we anticipated this task to be very difficult, we used the full images with peripheral information. In the task, J was relatively unimpaired, scoring 48% for naming of the blurred images and 85% for the unaltered images. Therefore, J’s performance is surprisingly high, even when configural processing is allegedly the only viable strategy. From discussions with J, we learned that she claims to be able to judge “*the interplay of different face parts”* when confronted with blurred images. While intact configural processing in DP has been reported before [43, 44], it raises the question of what mechanism can explain the deficits observed in DP and why the good performance even under adversarial conditions does not easily translate into everyday life. When asked about this, J claimed that one difficulty in everyday life is that familiar faces will not pop out from a crowd. However, they often can be found with enough effort. For example, while J might walk by her husband on the street, she might be able to find him at a designated spot. Similarly, J might scan a lecture hall row by row and seat by seat to finally find a fellow student she is looking for. This “*one face at a time”* strategy might also allow for good performance in certain experimental settings, like the present task.

#### The face’s social information

Another aspect of face processing that is usually unimpaired in people with DP is the recognition of emotions, intentions or other social information, such as trustworthiness [45, 46]. Despite her prosopagnosia, J says she is very good at picking up subtle social cues, which also aid her in deciding whether someone might be familiar: “*I can recognize if someone recognizes me; and then I act accordingly”*. She also judges her skills to tell if her interaction partner is lying, agitated or uncomfortable as very high: *“I think I have a very keen understanding of social signals. The way the eyes of a person change when they are uncomfortable, small pauses they make in their speech and certain subtle facial expressions”.* In order to objectively assess her ability to derive social cues from faces, we used the “Reading the mind in the eyes” test, which measures the ability to imagine the mental states of others [47]. In the test, only the eye region of a person’s face is shown and the participant is asked to select one word out of four which best describes the mental state of the depicted person (for example, “ashamed”, “alarmed”, “bewildered”, “irritated”). On the test, J scored 26 points out of 36 (72% correct), which is at the lower end of the normal range (cf. Table 1). The only other case of DP in the literature who performed the “Reading the mind in the eyes” test also showed poor performance, even significantly below the normal range [48].

**Fig. 5:**
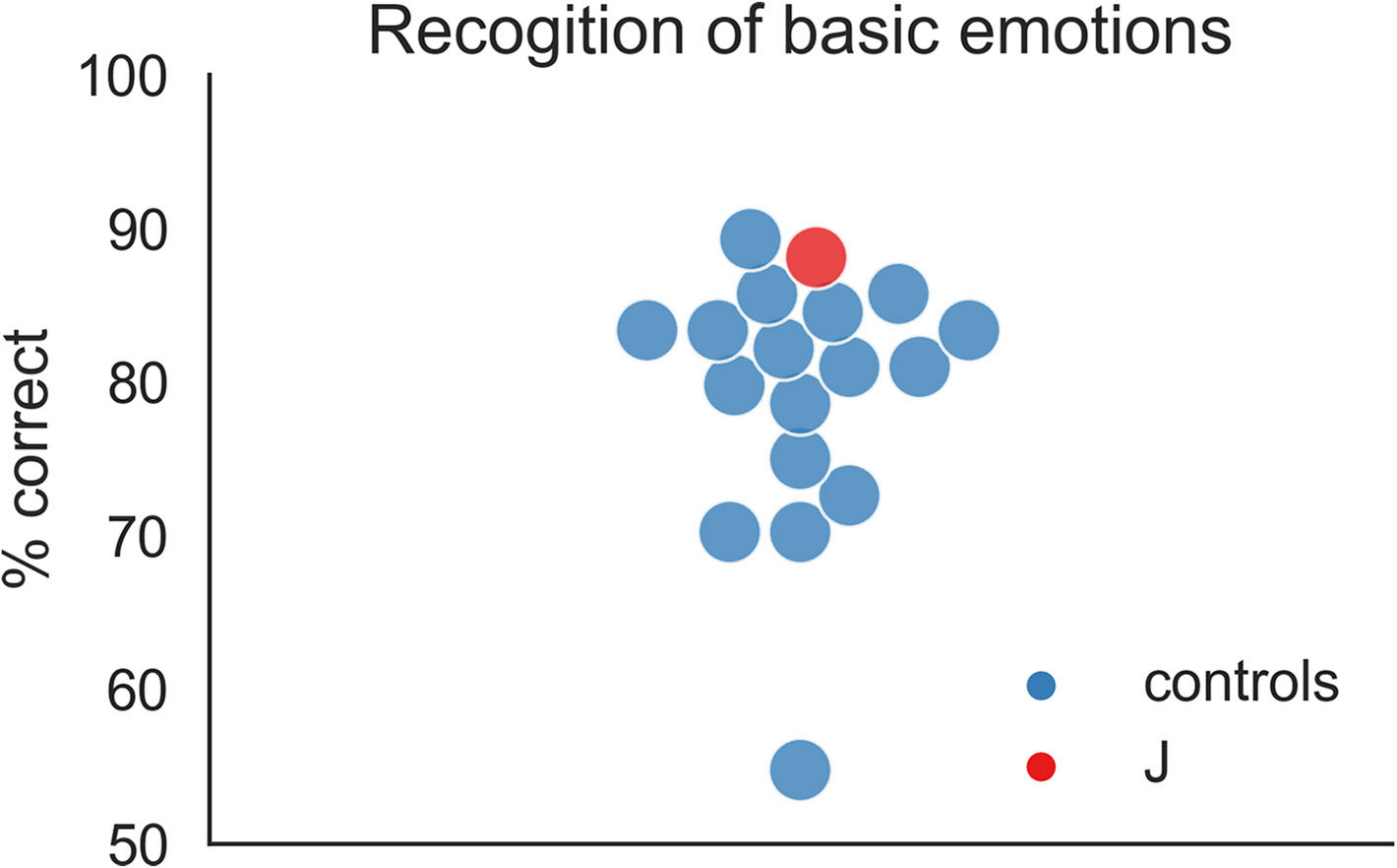
Results of the emotion recognition task. In the task, one face was shown at a time (happy, angry, fearful, sad, disgusted, surprised or neutral) and had to be labeled in a 7-way forced-choice decision. The figure shows the percentage of correct responses for 17 unmatched male control participants in blue and for J in red. J scores 88% correct, making her the second-best performing participant in the sample. Control data were taken from [51]; stimuli were taken from the NimStim database [52]

To further test J’s ability to identify facial expressions of emotion, we used a 7-way forced-choice basic expression recognition task, with happy, sad, angry, fearful, disgusted, surprised and neutral faces. Here, J scored 88% correct and was the second-best participant in our sample (Fig. 5). This is in line with most studies indicating that individuals with DP are unimpaired in recognizing emotions in faces [49, 50]; but see [48] for deficits in emotion recognition).

#### Attractiveness and likability of faces

In addition to identifying intentions and emotions, J is also convinced that she can reliably judge the attractiveness of faces, and a number of episodes from her life seem to corroborate this impression.

In one episode, J recalled being at a discotheque with a female friend. She saw a man she found handsome, had a drink with him and they talked for some amount of time. The other day, she was at a café with the same friend, as a man walked by. She remarked to her friend that he also looks quite handsome, to which her friend replied that this was the same man she had drinks with the night before. The same thing happened a third time, only a few days afterwards, with the same man, in pretty much the same way. This provides anecdotal evidence that J’s judgments of attractiveness are consistent across time.

J also reported that she likes *“odd”* faces and takes an immediate liking to anyone with some idiosyncratic features in their face. She finds people with green eyes or thick eyebrows immediately likable, predominantly because they are easier to identify and consequently interaction with them is considerably less stressful. *“I like red hair a lot and take an immediate liking to anyone who has it”,* J told us. Similarly, other cases of DP described in the literature have remarked that they focus on the “worst features” of each acquaintance’s face to remember them, keep company with “physically distinctive” people and claim that forgetting someone’s face should be regarded a compliment, because good looks are not memorable [9].

**Fig. 6:**
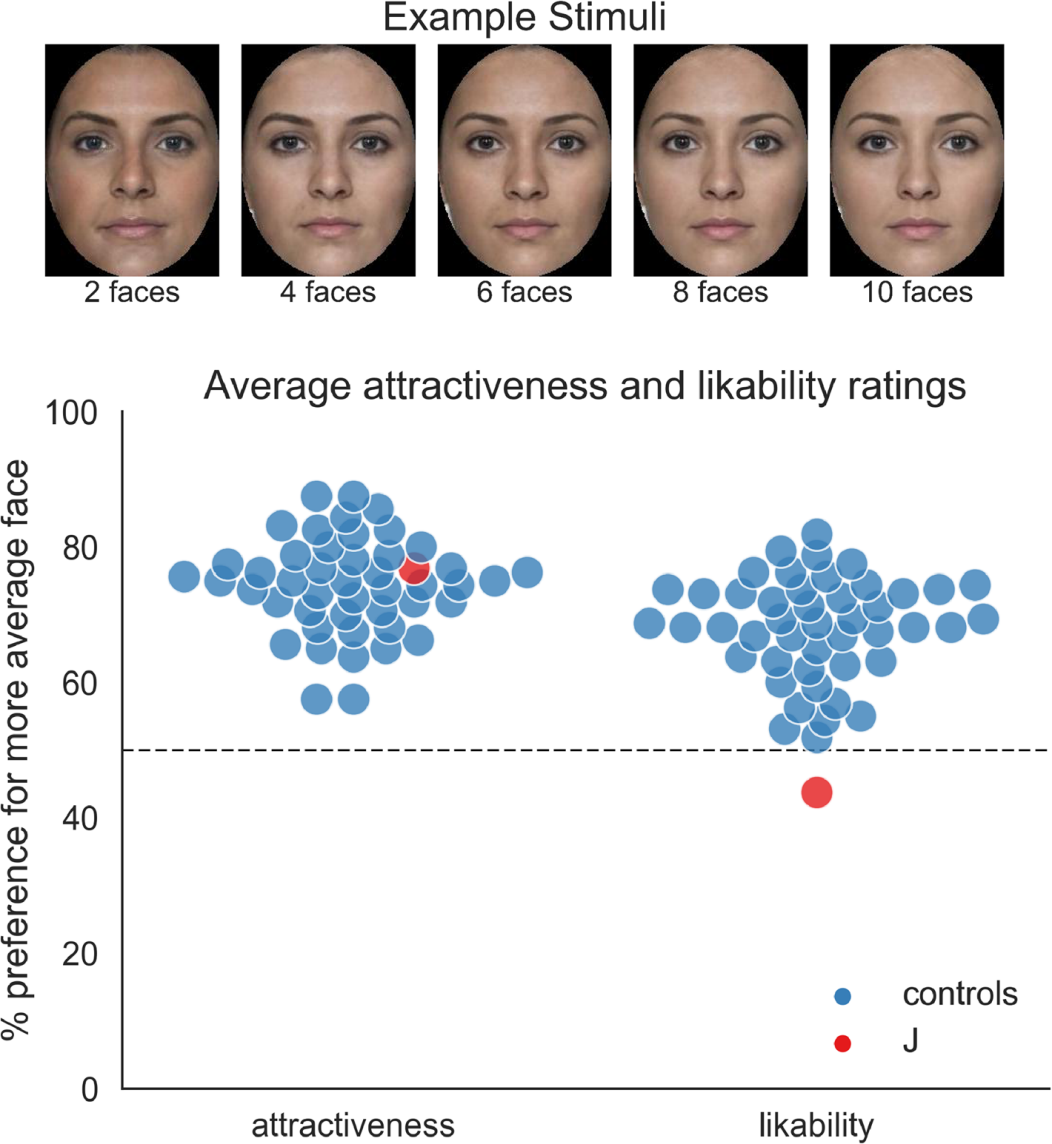
Results of the average faces task. The upper part of the figure shows a set of example stimuli, with the number of faces that were averaged to create the stimulus increasing from left to right. In the task, a random pair of faces was shown in each trial and the participants had to decide which one is more attractive (part 1) or more likable (part 2). The lower part of the figure shows the percentage of preferences for the more average face. If the average faces are preferred, the responses will lie above 50%. If the less average faces are preferred, the responses will lie below 50%. A response at exactly 50% will indicate absence of a systematic preference of one over the other. Responses for 46 female control participants are shown in blue and results for J are shown in red. Stimuli were created using faceresearch.org. Stimuli are based on images from DeBruine & Jones, available under a CC-BY licence from 10.6084/m9.figshare.5047666.v3

**Fig. 7:**
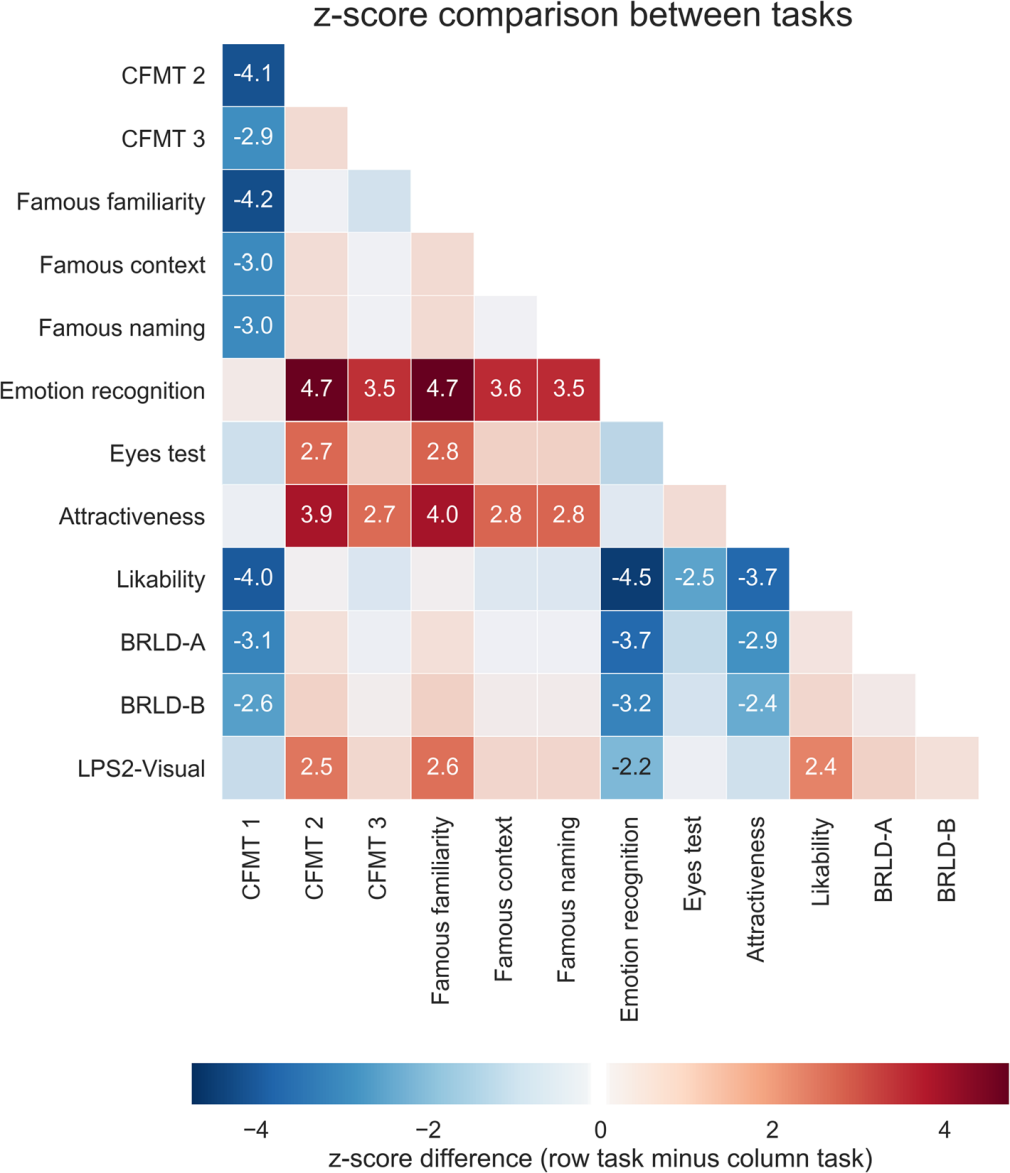
Comparison of J’s performance for all major tasks. Differences in z-scores for all pairings of tasks are reported, with positive scores indicating higher performance for the tasks listed in the rows, compared to the tasks listed in the columns. Differences larger than two standard deviations are highlighted by stronger colors, with the given annotations indicating the numerical difference in z-scores. The emerging pattern suggests that J’s performance is impaired for tasks involving learning of multiple faces (CFMT 2 and 3), and the recognition of famous faces from their inner parts, compared to learning of a single face (CFMT 1) and the recognition of emotion, intention (Eyes test) and attractiveness in faces. CFMT: Cambridge Face Memory Test; Eyes test: Reading the mind in the eyes; BRLT: Bergen Right-Left Discrimination Test; LPS2-Visual: Leistungsprüfsystem 2 (“performance test system”, test of visuo-spatial skills)

Increasing the averageness of a face normally increases its perceived attractiveness [53]) while making it less distinctive. Accordingly, we prepared a task where faces with different degrees of averageness were presented (Fig. 6). In each trial a pair of faces was shown, one more and one less strongly averaged, and J had to make a choice which one she finds more attractive or more likable. Compared to a group of 46 female control participants, J showed a prototypical preference for more average faces when it comes to attractiveness, but was the only person in the group to find less average faces more likable. The results show that in DP, ratings of attractiveness and likability might dissociate (Fig. 6, Fig. 7). The findings are both in line with studies showing normal attractiveness ratings for individuals with DP [54], as well as the until now incompatible observation that they report liking ‘odd’ faces more.

## Discussion and conclusions

We have presented the case of J, a 30-year old woman suffering from DP, a lifelong face recognition deficit. J presents with a pure form of DP, showing strongly impaired recognition of identity for both unfamiliar and familiar faces, but no difficulties recognizing expressions, intentions or attractiveness from faces (cf. Table 1, Fig. 7). She can also recognize identity from uncropped images of faces, indicating preserved configural processing of faces with peripheral features.

The pattern of J’s deficits lends further support to the notion that face processing is special, in that it can be dissociated from the processing of other objects [55], and that recognizing identity from faces can be independent from recognizing other aspects of the face, such as emotion or intention [56, 57].

While DP certainly is a heterogeneous disorder [5, 44] and it is difficult to generalize from the single case, the CFMT provided the most unequivocal results in the present study, as it allowed us to best delineate J’s performance from the control participants. The perfect performance on the first part of the CFMT, where only one face has to be learned and recognized, also suggests that J’s difficulties cannot be due to problems with face perception. This part of the CFMT strongly dissociated from the other two parts, where multiple faces have to be learned and recognized (Fig. 7). In line with the present results, the CFMT has been successfully used to identify DP in a number of previous studies (e.g. [38, 48]). J also told us that she found the CFMT to reflect her problems most faithfully, because many faces need to be learned at once and even extreme conscientiousness did not allow her to perform well when using only single features. A test that has so far worked for the majority of patients in the literature is important, especially to improve the state of clinical diagnostics in DP [14, 15]. However, while perfect performance on the first part of the CFMT indicates that J likely has no problems with simple face matching, most people with DP have some problems in this part of the test [38]. Therefore, to better differentiate between face perception and face memory deficits when diagnosing DP, the CFMT should be accompanied by a face matching task, like the Cambridge Face Perception Test [7].

In contrast to the CFMT, the famous faces test showed less specificity, as some of the healthy controls performed very poorly on the task. This poor performance might be due to a cursory familiarity with the respective people (i.e., having seen one movie with an actor, as opposed to watching a show every week). Also, participants who know only a few famous people are more likely to be outliers, as knowing 40 people and scoring all correct or all incorrect is much less likely than knowing only 4 people and scoring all correct or incorrect. Hence, using faces that are completely new to all participants, as is in the CFMT, might make results more straightforward to interpret and compare.

Regarding explanatory mechanisms for J’s deficits in unfamiliar and familiar face recognition, we expected that impaired configural processing might offer the best explanation. However, the present study as well as some previous work in the literature has found largely intact configural processing in DP [43, 44]; but see [58] for contrary results). As we used uncropped images of faces including the peripheral features, one important question is whether testing configural processing of only the inner features of the face would have produced different results. Given the specificity of the disorder, it is reasonable to assume that in DP configural processing for other information (e.g. peripheral features) might be intact, and is impaired only for the inner features of the face. Indeed, two studies which investigated more general configural processing mechanisms found no deficits in DP [43, 44], while studies which used only the inner part of faces did find individuals with DP to be impaired [59, 60]. However, the fusiform gyrus is one potential neural correlate of the configural processing of faces [61], and individuals with DP have mostly shown prototypical activity patterns [29–31]. In line with these observations, J showed no structural or functional abnormalities in her fusiform gyri, at least on a macroanatomical scale. Therefore, the deficits in DP might manifest themselves higher up the face processing stream, for example in the anterior temporal areas responsible for matching a face to a person’s identity [62, 63]. These areas might constitute the neural basis for an impaired matching of novel face views with previously learned faces [43] in DP. Recognizing previously learned faces is highly variable in DP, and previous studies with famous faces already showed that hit rates were higher than would be expected from individuals who cannot recognize their parents or spouse [16, 41]. For the first time, we showed that above-chance performance of individuals with DP in famous face tasks is not necessarily due to flukes or a function of familiarity. We hypothesize that these instances of successful recognition can be due to simple featural strategies that are not supposed to work for identity recognition, but sometimes do work nonetheless. For example, J was able to recognize Mr. Obama by using a rare but simple-to-memorize combination of local features, like freckles on a dark complexion. Also, sometimes information from a different face-processing pathway, for example the pathway of processing expressions [56, 57], might be successfully exploited for identity recognition. For example, J was able to recognize Ms. Merkel by information that is not identity-related but nevertheless stable over time, like a subtle facial expression that is present on most pictures of her.

Given that under favorable circumstances a person with DP can recognize faces quite well, the results raise the question of why these abilities do not translate into everyday life. Among the most discriminative symptoms of DP are face recognition problems in “crowded places or out-of context encounters” [64]. J also claims that familiar faces do not pop out from a crowd but can be found “*one face at a time”*. This might indicate that tasks which more closely emulate situations in everyday life need to be developed. For example, the effect of using more complex visual stimuli with distracting or misleading peripheral information could be explored. This might allow a better understanding of the challenges individuals with DP face in their daily life.

That a better emulation of real-life experience in the lab is possible is shown by our investigation of perceived attractiveness and likability of faces. Here, the subjective experience of preferring distinctive faces, and lab results indicating typical attractiveness ratings, could be reconciled. The results have shown that while a person with DP might not have an altered sense of what makes a face attractive, they might take a stronger liking to more distinct faces. This subtle dissociation was absent in the non-DP persons we tested.

In summary, we present a pure case of DP, in which deficits are restricted to an impairment in recognizing people’s identities from their faces. By carefully delineating the deficit using experimental methods as well as detailed reports of J’s subjective experience, we show that neither lack of familiarity nor impaired configural processing in general can explain the deficits. J’s difficulties show most strongly when only the inner features of the face are visible and no single feature is specific for a given face. This suggests the conclusion that an inability specific to integrating the inner features of the face into a whole, might be the best explanation for the difficulties this and other individuals with DP frequently experience.

## Materials and methods

### Agnosia screening

To screen for visual agnosia, we used a set of full-color photos of 12 objects, cut-out along their contours. The images consisted of living objects such as animals, fruits or vegetables and of non-living objects such as tools, clothes or furniture. The screening consisted of 8 free-naming trials where a single image was shown and had to be named, and of four forced-choice identification trials, where an object was named and had to be selected from a configuration of four objects.

### Structural and functional MRI

All MRI data were collected using a 3 T Siemens Trio scanner. A high-resolution T1-weighted structural image was acquired with 0.75 × 0.75 in-plane resolution and 0.8 slice thickness (192 sagittal slices) using a 32-channel head coil. Task-based fMRI data were collected using a high-resolution EPI sequence with 2x2mm in-place resolution and 2 mm slice thickness (40 axially oriented slices; TR of 3 s, 12 channel head coil). Slices covered the whole temporal lobe but omitted most of the rest of the brain. The first 3 volumes of each fMRI run were removed to allow for signal stabilization. Stimuli were presented using PsychoPy2 [65] and back-projected onto a screen which the participant viewed using a mirror mounted on the head-coil. In the first localizer task, short movie clips with fearful faces were shown, alternating with videos of landscapes. This paradigm has been used in previous studies [66, 67]. There were 8 blocks per condition with each block lasting 30 s (10 volumes), making a total experiment length of eight minutes. In the second localizer task, pictures and videos of faces and hands were shown in alternating blocks, each block lasting 12 s, with 24 blocks per condition, resulting in a total experiment length of 9 min, 36 s. All analyses were performed in native space using Freesurfer 6.0 (www.freesurfer.net; [68]). Structural data were preprocessed using Freesurfer’s recon-all function and fMRI data were motion-corrected and smoothed with 5 mm, full-width at half maximum (FWHM). FMRI data were analysed using Freesurfer’s FSFAST and results were visualized using PySurfer and SurfIce.

### Bergen right-left discrimination test

Body perception and mental rotation of bodies were tested using the The Bergen Right-Left Discrimination Test (BRLD; [36]). The test requires to identify the left and right hand on stick figures, which is made difficult by presenting the figures from the front or the back, and sometimes presenting figures with their hands crossed (BRLD-A). This is followed by a variation of the test where all stick figures are shown upside-down (BRLD-B) [35]. A maximum score of 144 can be reached for each part. Normative values based on a sample of *n* = 174 adult participants (124 female, mean age 28 years) were taken from [35]. For the BRLD-A, the normative sample has a mean score of 110 and standard deviation of 25. For the BRLD-B, the normative sample has a mean score of 110 and standard deviation of 28.

### LPS-2

Visuo-spatial abilities were tested using the visuo-spatial tasks from the LPS-2 [37], which consist of mental rotation, counting the plains of geometrical objects and matching geometrical forms. A summary score for visual-spatial intelligence can then be computed, with the highest reachable score being 120. Normative values based on a sample of *n* = 129 adults, with mean 76 and standard deviation 18 were taken from the test manual [37].

### Cambridge face memory test

To evaluate the memory for unfamiliar faces, we used the CFMT. The test consists of three parts, (i) learning one face, (ii) learning six faces (iii) learning six faces degraded by noise [38]. The control sample consisted of 27 female participants (mean age 27, range 25–35) who were students at Bielefeld University and did not report any history of psychiatric or neurological disorders. These participants also served as controls for the famous face tasks, which are described below. The experiment was programmed and presented using PsychoPy2.

### Famous faces task

To evaluate the recognition of known faces, we used an in-house constructed famous faces test. We asked J to provide us with a list of celebrities she thinks she can reliably recognize when she sees them on television or in newspapers, of which she could name 14 (cf. Table 2). 28 other famous people were added, roughly matched in terms of gender, occupation and ethnicity to the list of J. Critically, we also selected people that we knew J should be familiar with, but failed to put on her list: For example, she named the characters of Barney, Marshall and Lily from the Sitcom “How I Met Your Mother” on her list, which led us to include the other protagonists, Robin and Ted, as well. In addition to the famous people, eight images of non-famous people were included to control for response tendencies. We selected five pictures per person, which were converted to grey-scale and cropped with an ellipse so that only inner features of the face were showing.

For each face, three consecutive questions had to be answered: 1. “Is the person familiar to me?”; 2. “What is the person’s occupation?”; 3. “What is the person’s name?”. When evaluating the name, we counted real and character names as correct. After the experiment was over, each participant was debriefed by showing each famous person’s picture in color and with peripheral features, with name and occupation stated below the image. For example, a full-color uncropped image of Leonardo DiCaprio was shown, accompanied by the following information: “Leonardo DiCaprio, Actor, Django Unchained, The Great Gatsby, Titanic”. Hence, if given a full image and name and occupation a participant still claimed to not know the person, her respective answers for the main experiment were discarded from the analyses. The experiment was programmed and presented using PsychoPy2.

### Filtered faces task

A test aimed to distinguish between featural and configural processing of faces was constructed by adapting the famous faces test with the use of spatial filters. If a face is low-pass filtered, the resulting smoothed image lacks the details needed for a featural processing strategy and instead the face needs to be recognized by the arrangement of the features (configural processing). The use of spatial filters to induce featural and configural processing strategies for faces has been demonstrated before [69]. In the present experiment, each image was either shown in grey-scale without filtering, high-pass filtered (leaving only the high frequencies of an image, i.e., the edges) or low-pass filtered (leaving only the low frequencies of an image, i.e., smoothing). In contrast to the famous face experiment, here also peripheral features of the face were shown. As the experiment consisted of three parts (one for each filter type), only 24 famous faces were used to keep the length of the experiment comfortable for the participants. The single parts of the experiment were shown in a blocked fashion with the fixed sequence: low-pass, high-pass, and no filter. The experiment was programmed and presented using PsychoPy2.

### Reading the mind in the eyes test

In the test, participants see the eye region of a single face and have to select one of four terms that best describes the mental state expressed by that face. A total score of 36 can be reached. To compute percentile scores, we used a normative sample of 50 female student controls from a previous study [47]. This group has a mean score of 28.6 and standard deviation of 3.2.

### Emotion expression task

To test participants’ performance in recognizing facial expressions we used a 7-way forced-choice decision task with happy, sad, angry, fearful, disgusted, surprised and neutral faces. The face stimuli were taken from the NimStim database [52] and each emotion was displayed by a total of 12 different actors. In the experiment, a single face was shown for up to four seconds, and the participants were asked to choose which of the seven possible emotion expressions it depicts. The control sample consists of 17 males (mean age 43, range 24–58), hence controls are not matched regarding gender. The paradigm and the control group for this task are described in a previous publication [51]. The experiment was programmed and presented using PsychoPy2.

### Attractiveness and likability ratings

To investigate the judgment of attractiveness and likability of faces depending on their averageness, we used an in-house generated task, using faces from faceresearch.org [70]. Face stimuli were generated as follows: First, two random faces (of the same gender) were selected and averaged together. Then, subsequently more and more faces were added to the average in steps of two, so that there was an image with two, four, six, eight and ten averaged faces. Each subsequent average contained the faces from the previous set, plus two. In this fashion, eight female and eight male-based series of averages were constructed. All images were then cropped with an ellipse to contain only inner features.

The experimental setup was as follows: Two faces from the same average sequence were presented side-by-side (i.e., average with two and average with four faces) and the participant was asked to judge which face looks more attractive (or, in a separate block, more likable). This was repeated for every combination of pairs and all 16 identities, giving rise to 160 trials per block. Whether the experiment started with the attractiveness or the likability decisions was randomized. 46 female controls (mean age 25 years, range 17–49) were used as a control group and tested online using a browser-based presentation tool.

### Statistical methods

For each test, the percent of correct responses was computed as the ratio of J’s score relative to the maximal achievable score. J’s z-score for each test was computed based on the control sample’s distribution of scores, which was transformed to have zero mean and unit variance. The z-scores were converted into percentile scores using a cumulative distribution function. Inferential statistics were computed using the t-test method developed by Crawford and Howell, as described in [71]. Two-sided *p*-values were computed based on the t-value and the n-1 degrees of freedom (n being the size of the control sample). All computations were implemented using in-house software written in Python 2.7. A heatmap visualizing the differences of J’s z-scores in all major tasks was created using Seaborn (seaborn.pydata.org).

## Abbreviations

BRLT: Bergen right-left discrimination test
CFMT: Cambridge face memory test
DP: Developmental prosopagnosia
fMRI: Functional magnetic resonance imaging
FSFAST: FreeSurfer functional analysis stream
FWHM: Full-width at half maximum
HPF: High-pass filter
LPF: Low-pass filter
LPS: Leistungsprüfsystem
MRI: Magnetic resonance imaging
